# Novel Computational Methodologies for Structural Modeling of Spacious Ligand Binding Sites of G-Protein-Coupled Receptors: Development and Application to Human Leukotriene B4 Receptor

**DOI:** 10.1100/2012/691579

**Published:** 2012-12-10

**Authors:** Yoko Ishino, Takanori Harada

**Affiliations:** ^1^Graduate School of Innovation & Technology Management, Yamaguchi University, 2-16-1 Tokiwadai, Ube, Yamaguchi 755-8611, Japan; ^2^Graduate School of Biomedical Sciences, Hiroshima University, 1-2-3 Kasumi, Minami-Ku, Hiroshima 734-8551, Japan

## Abstract

This paper describes a novel method to predict the activated structures of G-protein-coupled receptors (GPCRs) with high accuracy, while aiming for the use of the predicted 3D structures in *in silico* virtual screening in the future. We propose a new method for modeling GPCR thermal fluctuations, where conformation changes of the proteins are modeled by combining fluctuations on multiple time scales. The core idea of the method is that a molecular dynamics simulation is used to calculate average 3D coordinates of all atoms of a GPCR protein against heat fluctuation on the picosecond or nanosecond time scale, and then evolutionary computation including receptor-ligand docking simulations functions to determine the rotation angle of each helix of a GPCR protein as a movement on a longer time scale. The method was validated using human leukotriene B4 receptor BLT1 as a sample GPCR. Our study demonstrated that the proposed method was able to derive the appropriate 3D structure of the active-state GPCR which docks with its agonists.

## 1. Introduction

G-protein-coupled receptors (GPCRs), the largest family of membrane proteins (around 800 in humans), are involved in a variety of biological and pathological processes such as development and proliferation [1], neurological disorders [2], angiogenesis [3], and metabolic disorders [4]. GPCRs transmit molecular information from the extracellular side to the intracellular side of the cell, thus mediating many intracellular responses. Although nearly one half of currently marketed drugs target GPCRs [5], only 10% of these receptors have endogenous ligands [6]. Structurally, all GPCRs share an architecture formed by seven transmembrane (TM) helices connected by extracellular and intracellular loops. GPCRs can recognize structurally diverse ligands ranging from photons to ions, amino acids, small organic molecules, lipids, peptides, or proteins [7].

Due to limitations in the purification and crystallization of GPCRs, until recently only a limited number of three-dimensional (3D) structures of GPCRs have been resolved at high resolution by X-ray crystallography: rhodopsin [8], the *β*-adrenergic receptors (*β*_2_AR [9, 10], *β*_1_AR [11]), the adenosine A_2A_ receptor [12], and the opioid receptors (*κ* [13], *μ* [14]). The highly conserved general architecture including the TM helical bundle and some universally conserved residues allows predicting the 3D structure of unknown GPCRs by homology modeling, while using a few known GPCR structures as a template [15]. However, great cautions are needed when utilizing the homology-based models for detailed GPCR structural and functional annotations since helix kinks are often different in different receptors. Modeling these subtle distinctions, which is essential for ligand docking and screening, remains a major challenge [16]. In addition, since almost all known GPCR structures have been found in their inactive forms, the homology modeling has shown little power to practically predict the 3D structure of GPCRs in the active forms. It is known that the agonist-bound GPCRs take the active forms, whereas the antagonist-bound or inverse agonist-bound GPCRs take the inactive forms.

Ligand-induced activation of GPCRs results in multiple allosteric conformational changes that propagate throughout the receptor structure, ultimately triggering different signaling cascades [17]. In contrast to inactive states of GPCRs, no crystal structures of active states were available until 2011. Given the absence of experimental structural information, several investigators have applied computational strategies to predict activated models of GPCRs [18–22]. Therein, it is reported that applying an appropriate long-term molecular dynamics (MD) simulation after homology modeling could predict plausible 3D structures of activated GPCRs in view of the induced fit mechanism [21, 22]. However, this approach needs to impose appropriate structural constraints prior to the MD simulation, which requires both the skills and experience of an MD specialist. Moreover, MD simulations on ordinary personal computers are generally limited to timescales up to the microsecond order in long-term studies, yet structural changes from fitting-induced fluctuations require calculations on the millisecond order. Due to its time-consuming nature, it is virtually impossible to simulate all processes involved in structural changes using only MD on common computers. For this reason, currently no high-accuracy methods are available for predicting the activated structure of GPCRs.

This paper describes a novel method to predict activated GPCR structures with high accuracy on standard workstations, while aiming for the use of the predicted 3D structures in *in silico* virtual screening in the future. First, we have developed a new method for modeling thermal fluctuations in GPCRs by using combinations of fluctuations with different time scales. Based on this model, we propose a novel computational method to search for one of the best 3D structures of an activated GPCR.

## 2. Model and Framework

Proteins undergo thermal fluctuations of considerable magnitude. The time scale of thermal fluctuations in proteins ranges from femtoseconds to minutes, or even longer timescales [23]. In terms of spatial considerations, various levels of fluctuations exist, which include rapid motions within microspace (such as thermal oscillations between atoms and rotations of amino acid side chains), and relatively slow but large structural changes (such as local unfolding). Not all of these motions can be simulated because of the physical limits of currently available MD calculations. We therefore propose a new method for modeling GPCR thermal fluctuations, in which fluctuations are combined on different time scales. In this approach, we first simulate the oscillations and fluctuations of various atoms at the picosecond and nanosecond levels to obtain native structure within a short time span. We then simulate the thermal fluctuations of GPCRs in terms of their characteristic rotational motions around helical axes, which represent fluctuations of greater time scales. In our approach, some information on GPCRs that is known *a priori* is utilized. For instance, we know that TM helices are immobilized in a cell membrane, so their mobility is certainly low. Furthermore, this approach is based on the previous findings that when a GPCR is activated in a cell membrane, its structural changes occur by rotations around the axes of TM helices [24], and that especially TM3, TM5, and TM6 play an important role in the activation process in some GPCRs [18, 21]. Although this method does not guarantee the optimum from an exhaustive chemical calculation approach, it does have the clear advantage of reducing calculation load without sacrificing the quality of the solution obtained. We specifically focus on predicting the central part of the 7-TM helical bundle, a spacious ligand binding site, with good quality, while aiming for the use of the predicted 3D structures in *in silico* virtual screening in the future.

Based on this model, we propose a framework for structural search which comprises three stages, namely: (i) obtaining initial structures of a target GPCR by homology modeling using known GPCRs in their inactive forms as a template, (ii) obtaining average structures of a GPCR in a stable state after simulating fluctuations from several hundred picoseconds to a nanosecond using MD simulations, and (iii) obtaining a best 3D structure of a target GPCR in the active form through a machine-learning technique including evolutionary computation and modularized ligand-binding simulations. Our proposed method employs a real-coded genetic algorithm (GA) as an evolutionary computation to search appropriate rotation angles of helices of a target GPCR, in which discrete steps employ receptor-ligand docking simulations to evaluate the goodness of the rotation angles of the GPCR. 

The core idea of the framework is that an MD simulation is used to calculate the 3D coordinates of all atoms of a target GPCR in a relaxed state in terms of heat fluctuation on the picosecond or nanosecond time scale, after which the evolutionary computation is used to determine the best ligand-bound rotation angles of helices of a GPCR as a movement on a longer time scale.

## 3. Methodology to Predict 3D Structure of Activated GPCR 

The detailed procedure of the proposed structural search method is described as follows.

**Figure 1:**
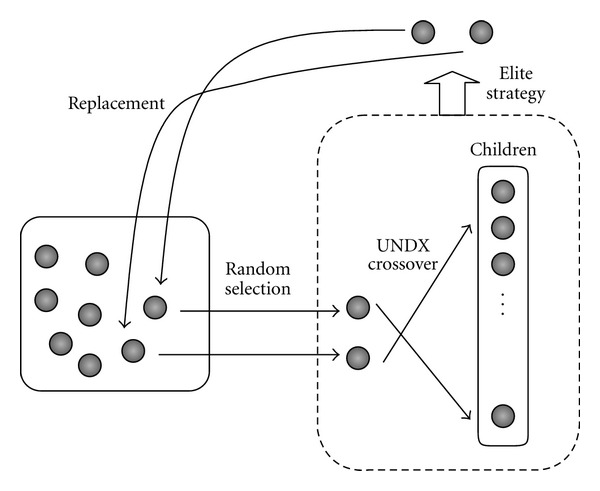
Schemes for generation alternation.

1. *Determination of an initial structure:* a known GPCR such as rhodopsin or beta-adrenergic receptor is used as a template. After alignment of the primary amino acid sequences of the template and target GPCR, the inactivated structure of the target GPCR is generated by regular homology modeling, which results in an initial structure.
2. *Average molecular structures with respect to fluctuations on a short-time scale:* an appropriate amount of water molecules are added to the surroundings of the target GPCR. The fluctuations of GPCR molecules are then calculated by MD simulations at the level of several hundred picoseconds. The average structures are defined as those for which the energy values stabilize.
3. *Structural changes with respect to rotational motions of helices:* hypothetical rotational motions around the axes of helices constituting the target GPCR (as fluctuations of greater-time scale) are generated. These are fed into a real-coded GA [25], as explained above, including simulations of binding with known ligands to determine optimal values. Details of the steps are described as follows. In addition, the schemes for generation alternation and offspring creation are shown in Figure 1.
  (3.1) *Generation of initial populations*. Individuals are assigned real-number vectors representing rotational angle changes relative to the average structures of various helices. These real-number vectors are treated as values within the space adjoining the two ends of the search space (i.e., toroidal space.) An initial population of *m* individuals is randomly generated.
  (3.2) *Creation of offspring for each generation*. Parents (two individuals) for random crossover of population pools are selected to match with a third parent, so as to generate offspring by the unimodal normal distribution crossover (UNDX) method [26]. The third parent is used to determine the standard deviation of normal distribution in UNDX. This procedure is repeated *n* times to provide *n* offspring individuals. In total, the number of individuals in a generation is *n* + 2 (offspring and two parents).
  (3.3) *Selection in each generation*. To evaluate each individual, receptor-ligand docking simulations are conducted using a known agonist and a known antagonist. An individual's real-valued vector translates into a corresponding 3D structure, with its side chains optimized before the binding simulations. An evaluation function has been established to represent how good the binding is. For a single individual, ligand-GPCR docking simulations are conducted up to *k* times, and each evaluation score is recorded. Upon obtaining the top score for each individual, the scores are then used to rank all individuals in a generation. The two individuals highest in ranking are selected (elite strategy) to replace the two parents in the population.
  (3.4) *Reiteration*. Steps (3.2) and (3.3) are repeated until some stop criteria are met.

The values for *m* and *n* can be determined based on previous researches and/or preliminary experiments. In our proposed search method, generation transition is achieved via the minimal generation gap (MGG) method [27]. MGG has good compatibility with UNDX and contributes to maintaining diversity in the population.

## 4. Test Case of Human Leukotriene B4 Receptor BLT1

This study reports on a novel approach to molecular modeling of 7-TM proteins that has been developed to build plausible active states of any given GPCR by using evolutionary computation. This heuristic approach starts from the amino-acid sequence of a target GPCR. We performed a validation test, where human leukotriene B4 receptor BLT1 was used as a sample target GPCR.

### 4.1. Leukotriene Receptor

Leukotriene (LT) is a bioactive lipid that serves as an important mediator of host defense, though it is also known to be implicated in bronchial asthma as a pathogenetic or precipitating factor. To date, four types of LT receptors have been cloned. One of these receptors, the high-affinity human LTB4 receptor BLT1 (GPCRDB entry ID: LT4R1_HUMAN; UniProt entry ID: Q15722), was selected as a target GPCR in our experiments. The length of the amino-acid sequence of BLT1 is 352aa.

### 4.2. Experimental Procedure

The sequence of BLT1 was retrieved from the UniProt database. ClustalX software [28] was used to align the sequence with the crystal structure of the bovine rhodopsin (UniProt entry ID: P02699) with which it shares a sequence similarity of more than 54%. One 3D structure of BLT1 obtained after running homology modeling using the bovine rhodopsin (PDB ID: 1L9H) as a template was selected from ModBase [29] as an initial rough 3D structure. ModBase is an open database of comparative protein structure models, theoretically calculated by the modeling pipeline ModPipe, which is maintained by Sali [30]. 

After annealing for atomic relaxation, MD simulations having distance restraints similar to a way proposed by Gouldson et al. [22] were run by using the TINKER software (ver. 4.2) [31]. The simulation conditions used were: force field = AMBER99; temperature = 310 K; pressure = 1 atm; time step = 1.0 fs. An average structure was determined after reaching a stable state, at which no steep drop in the molecular energy was observed. MD simulations for several hundred picoseconds were needed to achieve such a stable state.

The following are the processes of an evolutionary computation method in terms of optimizing the rotation angles of TM helices. The previous work performing amino-acid residue substitution analysis and spectroscopic experiments found that “TM3, TM5, and TM6 play an important role in activation of the leukotriene receptor [32].” This let us bound the free rotation space searched to these three helices; hence, the solutions we seek are the rotation angles of the three helices. Our proposed method employs a real-coded GA as a search algorithm and the ligand-binding simulation as an evaluation tool. An individual, which is defined as a real number vector representing the rotational angles of TM3, TM5, and TM6, has a unique value. In the evolutionary search process, the genetic operations mentioned in the previous section are repeated until the point when the stop criteria are met (the maximum generation number is 200, determined based on a preliminary experiment). The computational parameters in the evolutionary search are as follows: the initial population size is 50, and the number of offspring in a generation is 8. For evaluation of individuals, two sets of ligand-binding simulations are independently performed using a BLT1 agonist 12-keto-LTB4 (PubChem CID: 5280876) and a BLT1 antagonist pranlukast (PubChem CID: 115100). Docking of these ligands into the 3D structure of each individual, which is restored from the individual's vector representing the rotational angles of three helices to the 3D structure through structural relaxation by an *ab initio* (first-principle approach) computational method, is carried out with the GOLD software (ver. 3.1) [33]. The evaluation function in the evolutionary process is determined using the GOLD scores obtained from the docking simulations. The maximum GOLD scores derived from the docking with the BLT1 agonist and the BLT1 antagonist are assigned as *x*_1_and *x*_2_, respectively. The evaluation function value is shown below.

When *x*_2_ > 0, evaluation function value = *x*_1_ − *x*_2_; for all cases other than this condition, evaluation function value = *x*_1_.

Finally, a best BLT1 structure obtained through the proposed method is evaluated by executing the docking simulations with many ligands other than ligands used in the evolutionary search process.

### 4.3. Results and Discussion

**Figure 2:**
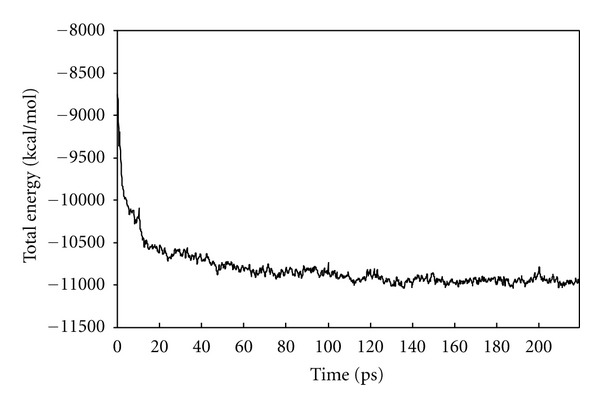
Molecular energy change of BLT1 through MD simulations.

A computer with an Intel Xeon 3.6 GHz CPU (dual processor) was used for computation. For MD processing of 219.3-ps simulations, 524 hours were required. We investigated the changes in GPCR molecular energy values (kcal/mol) for every 0.1 ps time elapsed in MD, as shown in Figure 2. Lower-energy values suggest corresponding increases in structural stability. At the point of approximately 200 ps, energy values ceased to further fall, which suggests that a region of stability had been reached. As our study focused on MD simulations for structural changes on a short-time scale (meaning that the objectives were to simulate structural relaxation), we considered 200 ps to be a sufficient level and therefore stopped at 219.3 ps. 

**Figure 3:**
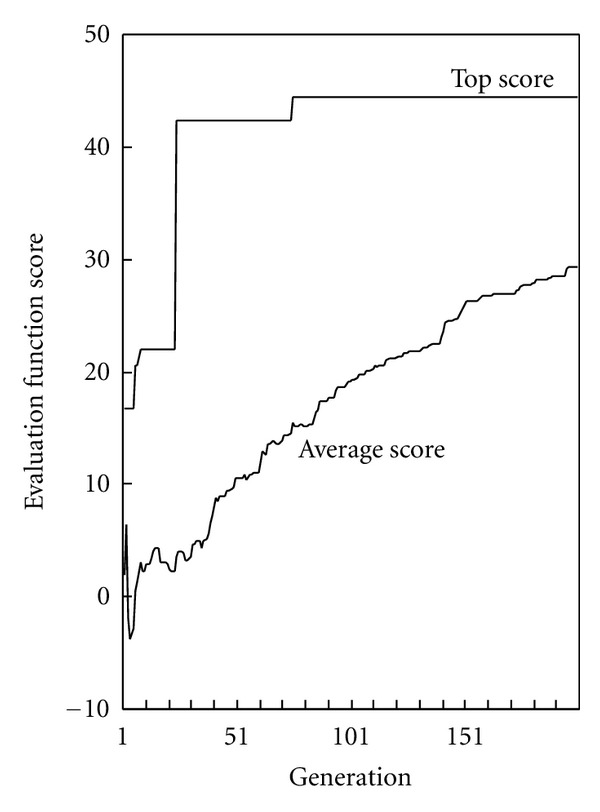
Evaluation function score in the evolutionary computation.

For subsequent evolutionary computation, 899 hours were required for calculations for 200 generations.  Figure 3 shows the trends of scores in evolutionary computation (the higher the scores, the better). The optimal solutions for rotational angles after 200 generations were: TM3 = 11°, TM5 = 14°, and TM6 = 255°.

**Figure 4:**
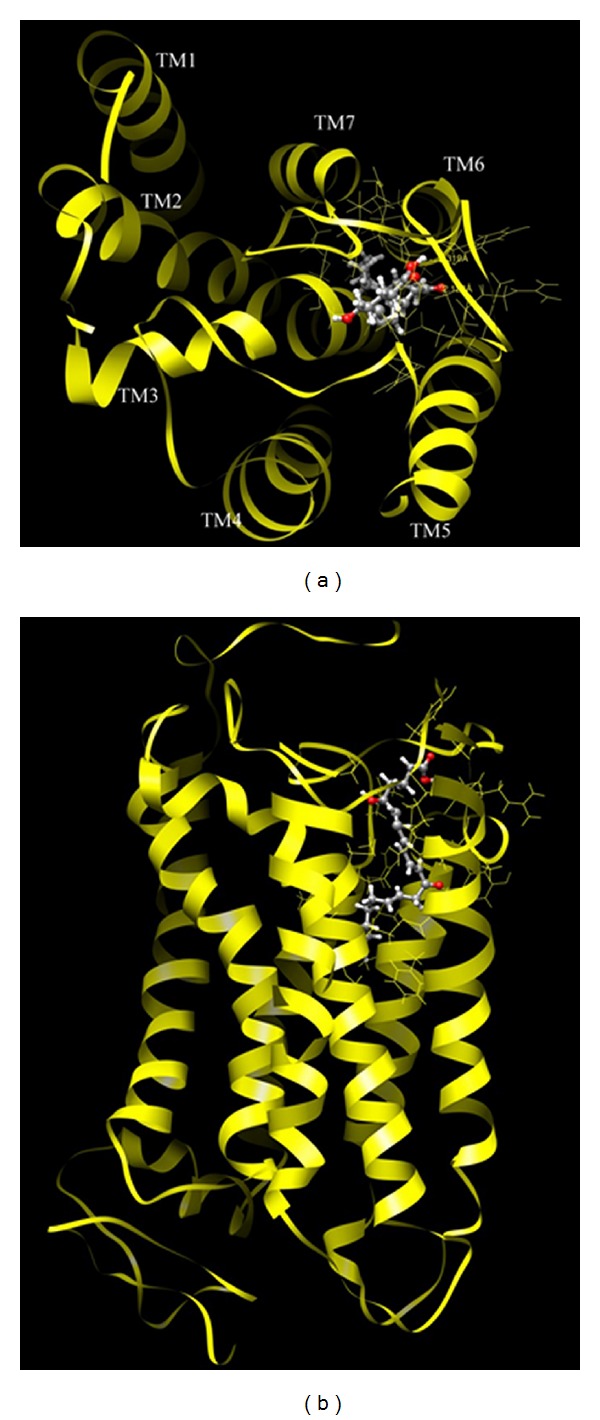
A best 3D structure of active-state BLT1.

Figure 4 shows the state of binding between the LT receptor and its agonist in one of the best 3D structures computed. The 3D structures of binding are shown in Figure 4(a) with a view from above the cell plasma membrane and in Figure 4(b) with a cross-section of the membrane (the upper part being the extracellular space). In order to highlight receptor-ligand interactions, the main chains (backbones) of the helices are shown as ribbons, while the BLT1 agonist 12-keto-LTB4 is drawn as a ball-and-stick structure. From Figure 4, it is clear that the BLT1 agonist binds to a recessed region (pocket) formed by TM3, TM5, and TM6. Upon further analysis regarding the binding state of the LT receptor and its ligand, these helices responsible for interacting with the LT ligand are composed mostly of hydrophobic amino acid residues, suggesting that the BLT1 ligand is drawn into a strongly hydrophobic environment. Since the BLT1 agonist has many hydrophobic groups, it is likely that ligand-receptor binding is contributed to mainly by hydrophobic interactions, though several hydrogen bonding sites contribute to the binding. The following interactions are worth detailing.

- Hydrophobic interactions between TM3 and the alkyl side chain near the central carbonyl (–C=O) group of the BLT1 agonist.
- Hydrophobic interactions between TM5 and the alkyl side chain near the central carbonyl group of the BLT1 agonist.
- Hydrogen bonding between the –OH group in the carboxyl (–COOH) group of the BLT1 ligand and the carbonyl group of Asn241 on TM6 (C=O⋯H–O intermolecular distance 2.32 Å.).
- Hydrogen bonding between the central carbonyl group of the BLT1 agonist and the main chain (–N–H peptide bond) of Gly246 on the loop joining TM6 and TM7 (C=O⋯H–O intermolecular distance 2.13 Å.).
- Hydrophobic interactions between TM6 and all alkyl side chains of the BLT1 agonist.

In addition to TM3, TM5, and TM6, we speculate that TM7 also contributes to hydrophobic interactions with the alkyl side chains of the BLT1 agonist.

At the beginning of evaluation, we calculated the root mean square deviation (RMSD) of BLT1 structures to investigate the structure change. Firstly, we obtained initial structures from homology modeling as structure I, MD relaxed structures as structure II, and structures generated from post-MD evolutionary computation as structure III to calculate the RMSD of 1,316 atoms forming the backbones. The resultant RMSDs were 4.49 Å for structures I and II, 1.55 Å for structures II and III, and 4.63 Å for structures I and III. Based on these values, the transition from structure I to structure II involved a large change. The reason why the transition from structure II to structure III was a smaller change is that the evolutionary search process only altered the rotation angle of TM3, TM5, and TM6. Nevertheless, what is interesting is that in the transition from structure II to structure III, the amount of rotational angle change in TM6 was clearly large compared with the other two TM helices: TM3 = 11°, TM5 = 14°, and TM6 = 255°. This is consistent with other previous research showing that the TM6 region plays a very important role in the transition from inactive-state to active-state rhodopsin [34]. Since GPCRs including the leukotriene receptor are allosteric proteins, the conformational change from inactive to active state likely affects the state of engagement with the associated G-protein. However, details of this mechanism require additional research.

**Figure 5:**
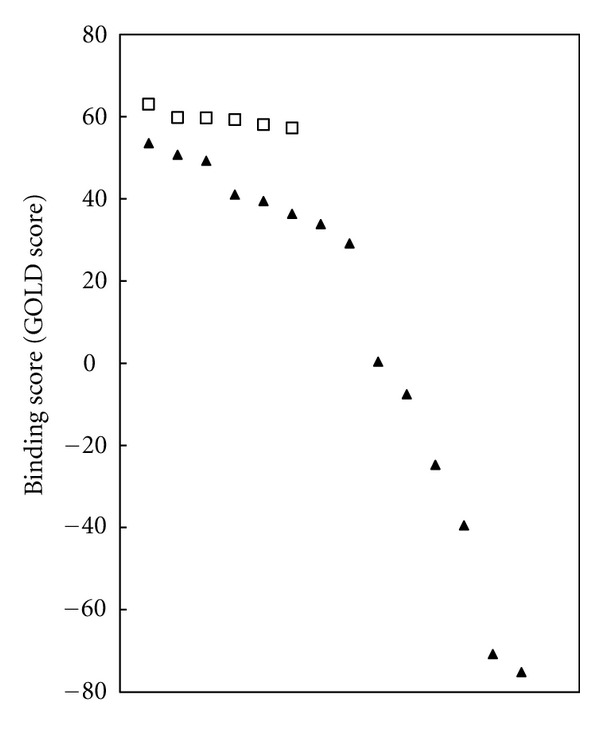
Ligand-receptor docking score (GOLD score) when docking with known agonists and antagonists of BLT1. Open squares show docking scores of known BLT1 agonists. Filled triangles show docking scores of known BLT1 antagonists. PubChem ID of BLT1 agonists (descending order): 5280492, 5280745, 5283156, 5280876, 5283129, 5280877. PubChem ID of BLT1 antagonists (descending order): 159476, 3081307, 6444688, 6439064, 6449854, 6540750, 132425, 6439436, 115100, 6442838, 204055, 177941, 196905, 192617.

In order to check the validity of LT receptor structures obtained by our method, we further used 6 known BLT1 agonists (including 12-keto-LTB4) and 14 known BLT1 antagonists (including pranlukast) to independently perform binding simulations with BLT1 of the optimal 3D structure, from which the corresponding maximum GOLD scores were determined. The results are as shown in Figure 5 such that the agonists and antagonists are plotted in order of decreasing scores. The agonist group produced scores that were visibly higher than those of the antagonist group. By using the Wilcoxon signed rank test (which makes no assumptions on normality of distribution), the GOLD scores of the agonist group and the antagonist group were found to be significantly different (*P* = 0.00046). This strongly suggests that the binding pocket obtained by our search method selectively binds with the BLT1 agonists.

**Figure 6:**
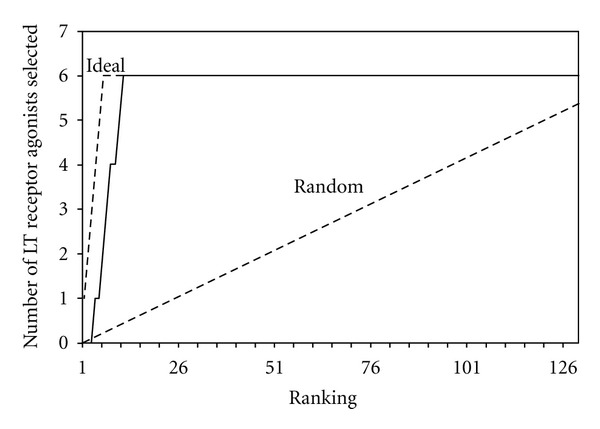
Enrichment curve for various GPCR ligands docked into the predicted active-state BLT1 structure.

Next, in order to assess the quality of the agonist binding states, we have challenged the best 3D structure model with a set of 144 GPCR ligands to determine whether the structure indeed shows a good preference for BLT1 agonists. The ligand set included 6 BLT1 agonists (including 12-keto-LTB4), 14 BLT1 antagonists (including pranlukast), and 124 other GPCR ligands (32 against serotonin receptors, 17 against histamine receptors, 17 against adenosine receptors, 14 against prostanoid receptors, 10 against dopamine receptor, 8 against adrenoceptors, 8 against angiotensin receptors, 7 against cannabinoid receptors, and 11 against other GPCRs), as shown in Table 1. Being GPCR ligands, these ligands automatically have “drug-like” properties, so the only filtering on the ligand selection was to ensure that BLT1- and non-BLT1 ligands had a similar molecular mass distribution. The ligand-binding simulations were individually performed using the GOLD software. The overall docking results for the most highly ranked conformations are shown in Figure 6. Based on the GOLD binding score (higher indicates better binding), all 6 BLT1 agonists were ranked in the top 11. The enrichment curve in Figure 6 shows that there is immediate enrichment, and the results are good compared to related studies [22, 35]. This also suggests that the binding pocket obtained by our search method selectively binds with the BLT1 agonists.

Consequently, we have developed a search method capable of predicting the 3D structures of active forms of GPCRs, which is broadly consistent with known facts of the target proteins and is reasonably persuasive as a model.

## 5. Discussion and Conclusions

With no crystal structures of active-state GPCRs other than mutant rhodopsin [34], it is indispensable to develop a practical method to predict 3D structures of activated GPCRs from their sequence information. In this study, we proposed a method for finding one of the best structures of an activated GPCR at a level of accuracy acceptable to virtual screening. The method was validated using human leukotriene B4 receptor BLT1 as a sample GPCR. Our study demonstrated that the proposed method including homology modeling, MD simulations, and evolutionary computing was able to provide the appropriate 3D structure of the activated leukotriene receptor to specifically dock with its agonists.

The first remarkable feature of this study is that the proposed method models GPCR thermal fluctuations on multiple time scales: the short-term heat fluctuation on the picosecond or nanosecond time scale and the rotational motions of TM helices on a longer time scale. This is a kind of coarse approximation compared with other methods like rigorous long-term MD simulations. However, the proposed method provides a significant reduction in computational cost when predicting the 3D structure of GPCRs and finally leads us to obtain the structure on common personal computers.

The second feature is that a machine learning technique including evolutionary computation and modularized ligand-binding simulations is employed for the structural prediction. Though to date, there have been methods using GAs to generate various conformations of ligands to explore a most suitable conformation in ligand-receptor docking [36], our method is the first approach using a GA employing a modularized ligand-binding simulation to find a best conformation on the receptor side.

However, some improvements might remain in the evaluation function of our evolutionary computing. Essentially, a scoring system capable of closely reflecting the binding-free energy would be desirable for the conformation evaluation. In our method, the GOLD scores produced by the GOLD software are directly used for the evaluation function. Although the GOLD scores are related with the values of the binding-free energy, they are not identical. We should accordingly investigate this issue in the future.

In addition, it should be noted that our method has a tendency to predict the central part of the 7-TM helical bundle, a spacious ligand binding site, with good quality. That is, small deviations would be observed in a spacious binding pocket area, whereas larger deviations would be observed in the distal section of the helices and even larger in the loop regions. This is because the evaluation function in evolutionary computing is naturally based on the ligand-binding score, closely related with the binding free energy. Hence, our method may predict another structure having energetically highly evaluated conformations of seven helices and the loops as a best structure if another search is performed. However, the structure of the spacious ligand-binding site would emerge again. We originally have the purpose of using the predicted 3D structure for virtual screening of candidate chemicals. Our method is able to provide 3D structure accurately enough for this purpose as shown in the case of the leukotriene receptor, since the structural information of the spacious binding pocket area is the most important for virtual screening.

We anticipate that parallelization of the proposed algorithm would contribute to an improvement in computation time. Future study designs will incorporate such. Furthermore, we plan to use GPCRs other than the leukotriene receptor to test the applicability and robustness of our search method. For such investigations, it would be important to carefully select an appropriate target GPCR. Based on sequence similarity within the seven TMs, GPCRs can be grouped into six families: the rhodopsin family A, the secretin family B, the glutamate receptor family C, the fungal pheromone family D, the cAMP receptor family E, and the frizzled/smoothened receptor family F [37]. In general, GPCRs belonging to class A fit the prediction, because all known GPCRs used as templates in homology modeling are classified into the class A. In contrast, other GPCRs having low amino-acid homology would need extra processing at the preliminary stage of homology modeling.

## Figures and Tables

**Table 1:** Ligands used for evaluation.

No.	PubChem ID	Receptor name	Ligand	GLIDA ID	Molecular weight
1	5280492	Leukotriene receptor	Agonist	L000354	336
2	5280745	Leukotriene receptor	Agonist	L000056	352
3	5283156	Leukotriene receptor	Agonist	L000050	320
4	5280876	Leukotriene receptor	Agonist	L000049	334
5	5283129	Leukotriene receptor	Agonist		336
6	5280877	Leukotriene receptor	Agonist	L000055	366
7	159476	Leukotriene receptor	Antagonist	L001349	304
8	3081307	Leukotriene receptor	Antagonist	L011298	414
9	6444688	Leukotriene receptor	Antagonist	L002037	502
10	6439064	Leukotriene receptor	Antagonist	L002263	360
11	6449854	Leukotriene receptor	Antagonist	L000610	361
12	6540750	Leukotriene receptor	Antagonist	L015882	346
13	132425	Leukotriene receptor	Antagonist	L006854	358
14	6439436	Leukotriene receptor	Antagonist	L009515	536
15	115100	Leukotriene receptor	Antagonist	L000493	481
16	6442838	Leukotriene receptor	Antagonist	L001488	455
17	204055	Leukotriene receptor	Antagonist	L006005	537
18	177941	Leukotriene receptor	Antagonist	L001468	544
19	196905	Leukotriene receptor	Antagonist	L006944	539
20	192617	Leukotriene receptor	Antagonist	L011726	600
21	68555	Dopamine receptor	Agonist	L013392	307
22	55483	Dopamine receptor	Agonist	L013398	356
23	5760	Dopamine receptor	Agonist	L000836	303
24	3341	Dopamine receptor	Agonist	L000254	306
25	5281878	Dopamine receptor	Antagonist	L000188	434
26	4748	Dopamine receptor	Antagonist	L000919	403
27	28864	Dopamine receptor	Antagonist	L000349	338
28	5265	Dopamine receptor	Antagonist	L000569	395
29	2818	Dopamine receptor	Antagonist	L000195	327
30	5074	Dopamine receptor	Antagonist	L001003	477
31	4038180	Adrenoceptors	Agonist	L000090	309
32	3251	Adrenoceptors	Agonist	L000768	581
33	13109	Adrenoceptors	Agonist	L000895	312
34	2419	Adrenoceptors	Antagonist	L000137	385
35	3372	Adrenoceptors	Antagonist	L000257	437
36	5640	Adrenoceptors	Antagonist	L000080	401
37	47811	Adrenoceptors	Antagonist	L000474	314
38	2369	Adrenoceptors	Antagonist	L000125	307
39	2790	Histamine receptor	Antagonist	L000192	308
40	55482	Histamine receptor	Antagonist	L000593	441
41	41376	Histamine receptor	Antagonist	L000313	321
42	3219	Histamine receptor	Antagonist	L001093	302
43	5533	Histamine receptor	Antagonist	L000771	371
44	65895	Histamine receptor	Antagonist	L000115	380
45	3947	Histamine receptor	Antagonist	L000944	418
46	2678	Histamine receptor	Antagonist	L000655	388
47	3957	Histamine receptor	Antagonist	L000667	382
48	2342	Histamine receptor	Antagonist	L000844	404
49	4830	Histamine receptor	Antagonist	L000727	375
50	475096	Histamine receptor	Antagonist	L000558	440
51	4940	Histamine receptor	Antagonist	L000807	340
52	4066	Histamine receptor	Antagonist	L000704	322
53	3348	Histamine receptor	Antagonist	L000869	501
54	124488	Histamine receptor	Antagonist	L001444	608
55	5282450	Histamine receptor	Antagonist	L001378	392
56	132059	Serotonin receptor	Agonist	L000336	411
57	127728	Serotonin receptor	Agonist	L000873	465
58	197706	Serotonin receptor	Agonist	L000746	403
59	57347	Serotonin receptor	Agonist	L000797	415
60	219050	Serotonin receptor	Agonist	L001062	346
61	3408722	Serotonin receptor	Agonist	L000428	365
62	4440	Serotonin receptor	Agonist	L000432	335
63	56971	Serotonin receptor	Agonist	L000682	401
64	91273	Serotonin receptor	Agonist	L000863	383
65	71351	Serotonin receptor	Agonist	L001106	420
66	5311258	Serotonin receptor	Agonist	L000364	351
67	5761	Serotonin receptor	Agonist	L000352	323
68	5311097	Serotonin receptor	Agonist	L000365	351
69	37816	Serotonin receptor	Agonist	L000794	340
70	3292447	Serotonin receptor	Agonist	L001346	486
71	16362	Serotonin receptor	Antagonist	L000494	461
72	3559	Serotonin receptor	Antagonist	L000288	375
73	55216	Serotonin receptor	Antagonist	L000503	346
74	4431	Serotonin receptor	Antagonist	L000685	393
75	5073	Serotonin receptor	Antagonist	L000510	410
76	2726	Serotonin receptor	Antagonist	L000182	318
77	55752	Serotonin receptor	Antagonist	L000720	440
78	5684	Serotonin receptor	Antagonist	L000012	422
79	28693	Serotonin receptor	Antagonist	L000396	403
80	4106	Serotonin receptor	Antagonist	L000397	356
81	68848	Serotonin receptor	Antagonist	L000394	362
82	37459	Serotonin receptor	Antagonist	L000016	361
83	107780	Serotonin receptor	Antagonist	L000275	497
84	3378093	Serotonin receptor	Antagonist	L000538	520
85	1229	Serotonin receptor	Agonist	L000011	321
86	3654103	Serotonin receptor	Agonist	L000142	406
87	4585	Serotonin receptor	Antagonist	L000455	312
88	133083	Angiotensin receptor	Antagonist	L008043	490
89	114899	Angiotensin receptor	Antagonist	L000467	480
90	3749	Angiotensin receptor	Antagonist	L000319	428
91	60921	Angiotensin receptor	Antagonist	L000533	610
92	2541	Angiotensin receptor	Antagonist	L000156	440
93	5281037	Angiotensin receptor	Antagonist	L000248	424
94	60846	Angiotensin receptor	Antagonist	L000621	435
95	5833	Angiotensin receptor	Antagonist	L001221	416
96	472880	Follicle stimulating hormone	Agonist	L000979	352
97	125672	Melanocortin hormone	Agonist	L000639	342
98	6434259	Prostanoid receptor	Agonist	L000194	424
99	6436393	Prostanoid receptor	Agonist	L000160	350
100	5311027	Prostanoid receptor	Agonist	L001211	415
101	5311503	Prostanoid receptor	Agonist	L000634	384
102	119304	Prostanoid receptor	Agonist	L000151	368
103	5311493	Prostanoid receptor	Agonist	L024041	350
104	6433212	Prostanoid receptor	Agonist	L000478	354
105	6439022	Prostanoid receptor	Agonist	L000570	362
106	122021	Prostanoid receptor	Antagonist	L000153	459
107	160	Prostanoid receptor	Antagonist	L000705	354
108	5311213	Prostanoid receptor	Antagonist	L024043	442
109	50294	Prostanoid receptor	Antagonist	L001326	371
110	132836	Prostanoid receptor	Agonist	L000098	388
111	5311035	Prostanoid receptor	Agonist	L000150	408
112	164437	Adenosine receptor	Agonist	L000290	388
113	104795	Adenosine receptor	Agonist	L000437	308
114	164305	Adenosine receptor	Agonist	L001390	363
115	93205	Adenosine receptor	Agonist	L000708	385
116	123807	Adenosine receptor	Agonist	L000165	369
117	3086599	Adenosine receptor	Agonist	L001283	499
118	5311506	Adenosine receptor	Antagonist	L000893	337
119	1329	Adenosine receptor	Antagonist	L000233	304
120	393595	Adenosine receptor	Antagonist	L000629	403
121	176408	Adenosine receptor	Antagonist	L000548	345
122	5697	Adenosine receptor	Antagonist	L000963	428
123	64627	Adenosine receptor	Antagonist	L001406	356
124	1970	Adenosine receptor	Agonist	L001216	399
125	9576912	Adenosine receptor	Agonist	L001516	393
126	122246	Adenosine receptor	Agonist	L000951	521
127	5287468	Adenosine receptor	Agonist	L000058	526
128	123739	Adenosine receptor	Antagonist	L001123	386
129	16078	Cannabinoid receptor	Agonist	L000023	314
130	5281969	Cannabinoid receptor	Agonist	L000111	347
131	5689	Cannabinoid receptor	Agonist	L000626	426
132	5488671	Cannabinoid receptor	Agonist	L000222	395
133	104895	Cannabinoid receptor	Agonist	L000004	376
134	39860	Cannabinoid receptor	Agonist	L000994	372
135	5311257	Cannabinoid receptor	Antagonist	L000363	383
136	4172142	Melatonin receptor	Agonist	L000306	348
137	4004	Melatonin receptor	Antagonist	L001138	330
138	5311198	Melatonin receptor	Antagonist	L000326	376
139	6024	Calcitonin receptor	Agonist	L000905	396
140	91498	Calcitonin receptor	Agonist	L024117	421
141	446284	Metabotropic glutamate group I	Agonist	L001256	302
142	6324636	Metabotropic glutamate group I	Antagonist	L000366	323
143	5311262	Metabotropic glutamate group I	Antagonist	L000372	383
144	123885	GABA-B subtype 1	Antagonist	L000172	401
